# Multidimensional cell-free DNA fragmentomics enables early detection of breast cancer

**DOI:** 10.1186/s13058-025-02190-8

**Published:** 2025-12-09

**Authors:** Lixian Yang, Mengyang An, Heng song, Xuan Zhang, Meiqi Wang, Liu Yang, Xinle Wang, Hua Yang, Xinyue Hong, Zhenchuan Song

**Affiliations:** 1 Breast Center, The Fourth Hospital of Hebei Medical University, 169 Tianshan Street, Shijiazhuang, 050000 Hebei People’s Republic of China; 2 Department of Breast Surgery, Xingtai People’s Hospital, No. 818 Xiangdu district, Xingtai, 054000 Hebei People’s Republic of China; 3 Department of Medical oncology, Hebei University Affiliated Hospital, Shijiazhuang, People’s Republic of China; 4 Geneseeq Research Institute, Nanjing Geneseeq Technology Inc, Nanjing, 210032 People’s Republic of China; 5 Key Laboratory for breast cancer molecular medicine of Hebei Province, Shijiazhuang, 050035 Hebei People’s Republic of China

**Keywords:** Breast cancer, Early detection, Cell-free DNA, Whole-genome sequencing, Machine learning

## Abstract

**Background:**

Cell-free DNA (cfDNA) fragmentomics represents a transformative approach for early breast cancer detection, offering significant potential to improve patient survival through timely intervention. Despite this promise, existing cfDNA-based methods demonstrate inadequate sensitivity for clinical implementation, particularly in early-stage malignancies. There remains an urgent need to develop robust, cost-effective diagnostic strategies integrating cfDNA fragmentomic profiling with advanced machine learning algorithms.

**Methods:**

This research involved a total of 191 participants who did not have cancer and 204 participants diagnosed with breast cancer. The plasma cfDNA samples from the participants underwent profiling through whole-genome sequencing. A variety of cfDNA characteristics and machine learning models were assessed within the training cohort to attain the best model. The evaluation of model performance took place in a separate validation cohort.

**Results:**

An assembled ensemble model that combines three cfDNA characteristics with six machine learning algorithms, developed in the training cohort (cancer: 119; healthy: 112), outperformed all models created from individual feature-algorithm pairs. This composite model demonstrated enhanced sensitivities of 93.3% at a specificity of 94.6% for the training cohort (area under the curve [AUC], 0.983) and 96.5% at 93.7% specificity for the validation cohort (AUC, 0.989) (cancer: 85; healthy: 79). Additionally, our model exhibited sensitivity across various stages, distinct pathological types, and diverse molecular classifications.

**Conclusion:**

We have established a stacked ensemble model using cfDNA fragmentomics features and achieved superior sensitivity for detecting early-stage breast cancer, which could promote early diagnosis and benefit more patients.

**Supplementary Information:**

The online version contains supplementary material available at 10.1186/s13058-025-02190-8.

## Background

Breast cancer persists as a formidable global health challenge, accounting for approximately 2.3 million new cases and 650,000 deaths annually, with hormone receptor–positive/HER2-negative subtypes representing the predominant burden [1]. According to the American Cancer Society, an estimated 313,510 new cases and 42,780 deaths are expected in the United States alone in 2024 [2]. Current diagnostic strategies include clinical examination, mammography, tissue biopsy, and blood-based assays for tumor-associated antigens or proteins [3, 4]. Early and accurate detection remains critical for improving patient outcomes, highlighting the urgent need for reliable, breast cancer–specific biomarkers to identify aggressive disease phenotypes [5].

Despite notable advances in breast cancer diagnosis and treatment, conventional approaches remain constrained by several limitations, including invasiveness, high cost, and suboptimal sensitivity and specificity, which may limit their applicability across diverse patient populations [6]. Standard diagnostic modalities such as imaging and tissue biopsy are further challenged by risks of false-positive and false-negative results, radiation exposure, and their inability to comprehensively capture the genomic and molecular heterogeneity of tumors [7–9]. These limitations underscore the urgent need for novel, noninvasive strategies capable of enabling early detection, predicting prognosis, and monitoring therapeutic response.

In this context, recent studies have increasingly highlighted the importance of circulating molecular biomarkers detectable in peripheral blood. Among these, circulating tumor cells (CTCs), microRNAs, and cell-free DNA (cfDNA) have attracted considerable attention due to their noninvasive nature and reproducibility in liquid biopsy assays. The ease of obtaining these biomarkers, coupled with their potential for real-time monitoring of tumor dynamics, has facilitated their application in early detection and therapeutic management [10–13]. These biomarkers hold promise as both diagnostic and prognostic tools, providing insights into treatment response, drug resistance, and metastatic progression [14–16]. Moreover, accumulating evidence suggests that circulating biomarkers may play a pivotal role in advancing personalized medicine strategies for breast cancer patients [11, 14, 16–19].

Cell-free DNA (cfDNA) consists of short segments of nucleic acid found in plasma. At first, it was thought that these fragments originated from processes such as apoptosis or necrosis [20, 21]. Nonetheless, recent research suggests that cfDNA may be actively secreted by tumor cells [22–27] and might also be associated with circulating tumor cells (CTCs) present in the bloodstream [28]. A significant portion of studies on circulating cfDNA has focused on its potential as a liquid biopsy biomarker, either for assessing tumor burden through concentration analysis or for monitoring treatment response and prognosis by investigating genetic and epigenetic modifications. Techniques centered on methylation, such as whole-genome bisulfite sequencing and additional bisulfite modifications in next-generation sequencing (NGS) approaches, have emerged as promising methods for the early identification of cancer. Nonetheless, these techniques are associated with significant drawbacks, including procedural intricacies and prolonged timelines. As a result, there is an urgent need for other strategies that can mitigate these technical challenges and reduce the time required, while maintaining high sensitivity and specificity for the early detection of cancer.

The emerging field of fragmentomics, which investigates the patterns of circulating free DNA (cfDNA) fragmentation, is anticipated to address the existing gap caused by the insufficiency of comprehensive and detailed whole genome sequencing (WGS) data. Notably, the fragmentation patterns of cfDNA exhibit a heterogeneous distribution across the genomic landscape. These patterns are believed to be influenced by chromatin structure, offering essential insights into nucleosome arrangement and gene expression [13]. A notable breakthrough was achieved with the DNA Evaluation of Early Intercepted Fragments (DELFI), which assesses the coverage, size, and various summary statistics of fragments within a 5 Mb region [29]. The ratios of short (100–150 bp) and long (151–220 bp) cfDNA fragments were found to differ between healthy individuals and cancer patients, allowing for differentiation according to disease status. Furthermore, flagmenomics studies have integrated additional methods, including preferential terminal coordinates [30] and terminal motifs [31], to differentiate between cancerous and non-cancerous groups. Recently, analyses of transcription factors linked to cfDNA have been conducted to identify specific patterns unique to patients and tumors with significant accuracy [32].

Recognizing the valuable insights provided by circulating free DNA (cfDNA) and its capacity to distinguish between cancerous and non-cancerous states, we developed a model designed to encapsulate the intricate landscape of cfDNA. By utilizing five refined fragmentation attributes, we employed a robust integrated stacking machine learning methodology to construct the model. The primary objective of this study is to present a model that is both highly sensitive and economically viable for the identification of breast cancers (BCs), potentially leading to significant clinical benefits in real-world medical applications.

## Materials and methods

### Participant enrollment

For model construction and internal validation, we enrolled 395 participants in this study cohort, including 204 patients with breast cancer (BC) and 191 healthy participants, all sourced from the Fourth Hospital of Hebei Medical University. The present study was approved by the Medical Ethics Committee of the Fourth Hospital of Hebei Medical University (No. 2023KY032), and was carried out in accordance with the Declaration of Helsinki.

### Whole genome sequencing for plasma samples

Ten milliliters of peripheral blood were drawn from participants into EDTA tubes. This was followed by centrifugation at 16,000 x g for a duration of 10 min within six hours to isolate the plasma. The resulting plasma samples were then stored at a temperature of -80 °C until they were transported on dry ice to the central laboratory at Nanjing Geneseq Technology Inc., a clinical testing facility located in China.

Upon arrival, the plasma samples were stored temporarily at -20 °C for roughly 1 to 3 days, depending on when the sequencer became available. Cell-free DNA (cfDNA) was isolated from the thawed plasma samples utilizing the QIAamp Circulating Nucleic Acid Kit (Qiagen). The cfDNA concentration was subsequently assessed with the Qubit dsDNA HS Assay Kit (Thermo Fisher Scientific), following the protocols outlined by the manufacturer. Following this, 5–10 ng of cfDNA was employed to construct whole genome sequencing (WGS) libraries, utilizing DNA end repair, A-tailing, and adapter ligation with the KAPA Hyper Prep Kit (KAPA Biosystems; according to the manufacturer’s guidelines). The resulting libraries were quantified using KAPA SYBR FAST qPCR Master Mix (KAPA Biosystems) prior to being sequenced in paired-end mode on the T7 platform (MGI).

To maintain the integrity of the data, a thorough array of quality assurance procedures was implemented on the generated sequence information. The first procedure included the refinement of sequence reads with Trimmomatic (v0.36.6). Next, PCR duplicates were identified and removed using the Picard toolkit (http://broadinstitute.github.io/picard/). Once the trimming was completed, the sequences were aligned to the human reference genome (GRCh37/UCSC hg19) using the BWA aligner. QC metrics included Q30 scores, GC content, alignment rate to the reference genome, median insert size, and mean depth. Samples with sequencing QC failures (mean depth < 5×) were excluded. To ensure unbiased processing, the sample-operating team remained blinded to the case/control status throughout the workflow.

To address the variability in sequencing depths across samples, those with a depth of greater or equal to 5× were down-sampled to a uniform depth of 5× in order to optimize the model. The optimized model was subsequently validated using raw WGS data and additional down-sampling to depths of 4×, 3×, 2×, and 1× with the Picard toolkit, enabling a comprehensive evaluation of its performance.

### CfDNA fragmentomics features extraction

We utilized three distinct fragmentomics feature derived from cfDNA shallow WGS data, including: *C*opy *N*umber *V*ariation (CNV), *F*ragment *S*ize *D*istribution (FSD), and *FRAG*ment based *M*ethylation *A*nalysis (FRAGMA). The total number of extracted features reached 3,432, including 2,475 for CNV, 936 for FSD, and 21 for FRAGMA.

The extraction of CNV features was conducted according to the methodology described by Wan et al. [33], utilizing the ichorCNA tool. The genome was segmented into 1 Mb non-overlapping bins (totaling 2475 segments) based on the GRCh37/UCSC hg19 reference genome. The analysis incorporated multiple normalization steps: (1) GC content correction using precomputed GC wig files to address sequencing biases, (2) mappability adjustment with genome-specific map wig files to account for alignment artifacts in repetitive regions, and [20] systematic noise reduction through a Panel of Normals (PoN) constructed from healthy donor cfDNA samples to eliminate technical artifacts from library preparation and sequencing platforms. Log2 ratios were computed after these corrections, with autosomes serving as the diploid baseline (log2 ratio = 0). Regarding the details, we first use the readCounter tool from the HMMcopy suite (v0.1.1) to calculate the read counts in different regions of the genome. Then, we use runIchorCNA.R to correct for GC content and mappability, thereby obtaining the final CNV results.

For the **F**ragment **S**ize **D**istribution (FSD), we adapted the methodology from Cristiano et al. [29]. The FSD profile was generated by analyzing cfDNA fragments within the 110–220 bp range at 5-bp intervals (24 size bins per chromosome arm), covering 39 chromosome arms (totaling 936 features) while excluding the short arms of five acrocentric chromosomes (13p, 14p, 15p, 21p, 22p) due to limited sequencing coverage. Raw fragment counts were first corrected for GC-content bias using LOESS regression, then standardized to z-scores [(x-µ)/σ] within each size bin across all samples, where x represents the GC-corrected fragment count, µ the mean, and σ the standard deviation. This dual normalization approach effectively removed technical artifacts while enhancing biologically relevant fragmentation patterns for improved discrimination between cancerous and noncancerous cases.

The FRAGmentomics-based Methylation Analysis (FRAGMA) was introduced by Qing Z. et al. utilized cfDNA cleavage patterns around cytosine-phosphate-guanine (CpG) sites [34]. This methodology argued that an elevated likelihood of cfDNA cleavage adjacent to cytosine is indicative of methylation at CpG sites, whereas such a pattern is generally absent at unmethylated sites. Alu regions were specifically targeted where methylation levels are notably higher compared to the whole genome and CpG islands. Within these Alu regions, the fragment ratios of 5′ end motifs of CGN and NCG (N represents any nucleotide) resulted in eight fragment ratios. Additionally, the ratio of CGN to NCG motifs were computed. Notably, an enhanced rate of cleavage at the two cytosine positions was observed in CGCG sequence. Expanding this analysis, every pair of CpG nucleotides within the CGCG sequence was examined. The resulting fragment ratios were derived from 5’ end motifs of CGC, NCG, CGN, and GCG identified, resulting in ten fragment ratios for the CGCG sequence. Finally, the motif ratios of CGC/NCG and CGN/CGC were calculated.

### Machine learning model construction

The dataset used for training included a total of 119 patients with breast cancer and 112 healthy individuals, while the independent validation dataset was kept separate from the training process and was utilized solely for assessing the final model.

To construct predictive models, the input data for the model consisted of 3432 distinct feature columns (CNV: 2,475, FSD: 936, FBM:21) without further feature selection, ensuring that all available cfDNA characteristics contributed to both the cancer classifier model and the tumor of origin classifier model. During the modelling process, each fragmentomics feature type was used to build individual base models by applying six machine learning algorithms – Generalized Linear Model (GLM), Extremely Randomized Trees (XRT), Deep Regression Forests (DRF), Deep Learning [35], Gradient Boosting Model (GBM), and eXtreme Gradient Boosting (XGBoost). These algorithms helped generate scores ranging from 0 to 1 as the cancer probability indicator. Higher scores implied greater probability of cancer. The final stacked ensemble model was created by averaging the cancer scores from all algorithms. The model training and stacking process were completed by H2O AutoML framework (v3.36.0.3) [36]. This approach employed a random grid search to automatically optimize comprehensive hyperparameters across models, including booster type, maximum tree depth, column sampling, minimum rows per leaf node, number of trees, and regularization parameters (reg_alpha, reg_lambda). Importantly, the model was trained exclusively on the training dataset, ensuring the integrity of the validation set until the final performance evaluation. After training the base models, H2O AutoML ranked all models on a Leaderboard based on their area under the curve (AUC)metrics. Notably, 5-fold cross-validation was applied to both the based models and the stacked ensemble model. The training cohort was divided into five subsets, with four used for training and one for validation in each iteration. The model robustness was then ensured by repeating this process across all subsets. Additionally, base model training limits and ensemble techniques were incorporated in our study to enhance generalizability. Following performance evaluation of all models, we randomly combined the combined features with the top AUC from 6 algorithms to obtain the best-performing stacked model. Then, this optimal model was used to predict each sample in the validation cohort. The cut-offs were determined by training cohort at 95% specificity for the cancer classifier.

### All statistical analysis

The ROC curves were created using the pROC package (version 1.18.5). We calculated the sensitivity [TP/(TP + FN)], specificity [TN/(TN + FP)], positive predictive value (PPV) [TP/(TP + FP)], negative predictive value (NPV) [TN/(TN + FN)], and accuracy [(TP + TN)/(TP + FP + TN + FN)], as well as the corresponding 95% confidence intervals, based on true-positive (TP), true-negative [9], false-positive (FP), and false-negative [33] values using the epiR package (version 2.0.75). All statistical analyses were performed in R (version 4.4.1).

## Results

### Participants’ disposition and characteristics

**Fig. 1:**
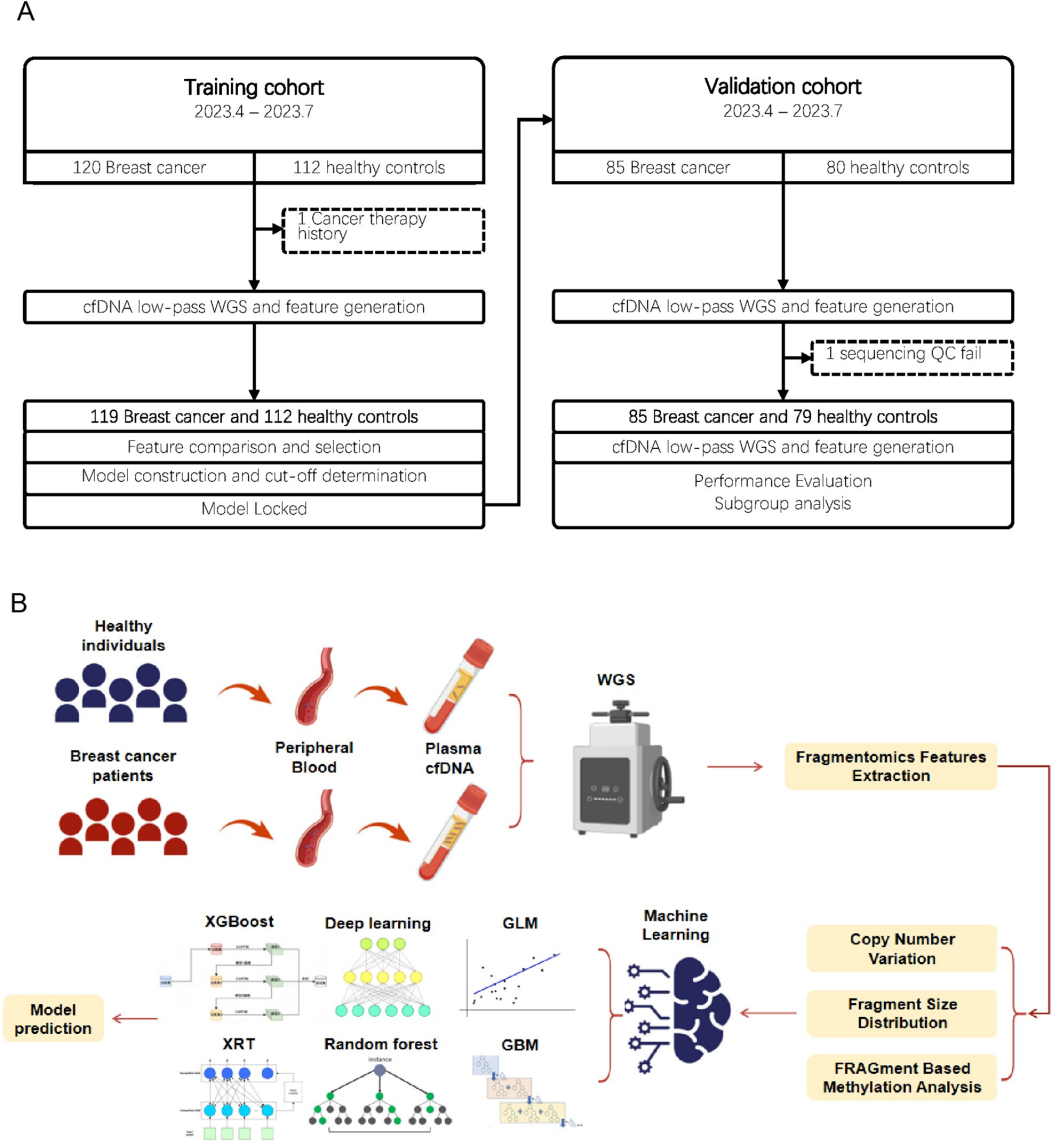
Dataset and model construction workflow **A **From 2023.04 to 2023.07, 119 breast cancer patients and 112 healthy participants were enrolled for the training dataset. To evaluated model performance, from 2023.04 to 2023.07, 85 breast cancer patients and 79 healthy participants were enrolled. **B** Schematic diagram of the stacked ensemble model construction and cancer probability score determination. Plasma samples went through the low-pass whole-genome sequencing (WGS) process, and the machine learning prediction model was constructed based on three cell-free DNA (cfDNA) fragmentomics features: Copy Number Variation (CNV), Fragment Size Distribution (FSD) and Fragment Methylation Analysis (FragMA). The genome-wide feature profiles were then applied to the six machine learning algorithms, with the resultant matrix processed by a second-layer GLM algorithm to form the stacked ensemble model and calculate the participant’s cancer probability score

The dataset utilized for training the model consisted solely of 231 individuals (119 BC patients and 112 healthy controls). For the validation cohort, 85 BC patients and 79 healthy individuals were included. Samples for this validation cohort were systematically gathered from the same center during the period of April 2023 to July 2023 (Fig. 1A).

Table 1 provides a summary of the demographics and clinical features for all participants in both the training and validation cohorts. In the training cohort, the average age of BC patients was 54 years, while in the validation cohort, the average age was 56 years. For healthy participants, the mean ages were 54 years in the training cohort and 55 years in the validation cohort. The cancer stage distribution remained similar between the cohorts, with most participants classified as stage II disease (training cohort: 52.4%; validation cohort: 50.6%). The participant group consisted entirely of females.

**Table 1:** Baseline characteristics of the sample population

		Training dataset		Validation dataset	
		Breast cancer	Healthy	Breast cancer	Healthy
		*N* = 119	*N* = 112	*N* = 85	*N* = 79
Age		56.2 [30, 79]	54.0 [45, 74]	52.7 [28, 74]	53.2 [45, 70]
Sex, percentage					
Female		119 (100%)	112 (100%)	85 (100%)	79 (100%)
TNM Stage, percentage					
Stage I		50 (42.0%)	N/A	23 (27.1%)	N/A
Stage II		59 (49.6%)	N/A	52 (61.2%)	N/A
Stage III		10 (8.4%)	N/A	10 (11.8%)	N/A
Histology type, percentage					
Ductal		99 (83.2%)	N/A	69 (81.2%)	N/A
Papillary		9 (7.6%)	N/A	4 (4.7%)	N/A
Lobular		4 (3.4%)	N/A	8 (9.4%)	N/A
other		7 (5.9%)	N/A	4 (4.7%)	N/A
Differentiation, percentage					
Moderate		83 (69.7%)	N/A	59 (69.4%)	N/A
Poor		27 (22.7%)	N/A	18 (21.1%)	N/A
Unknown		9 (7.6%)	N/A	8 (9.4%)	N/A
Molecular subtype, percentage					
HR- & HER+		77 (64.7%)	N/A	49 (57.6%)	N/A
HER+ (HR + or HR-)		30 (15.2%)	N/A	21 (24.7%)	N/A
TNBC		10 (8.4%)	N/A	12 (14.1%)	N/A
Unknown		2 (1.7%)	N/A	3 (3.5%)	N/A

Overall, the demographic characteristics showed similarities between the training and validation cohorts, ensuring reliable performance estimates.

## Feature assessment and ensemble stacked model evaluation

**Fig. 2:**
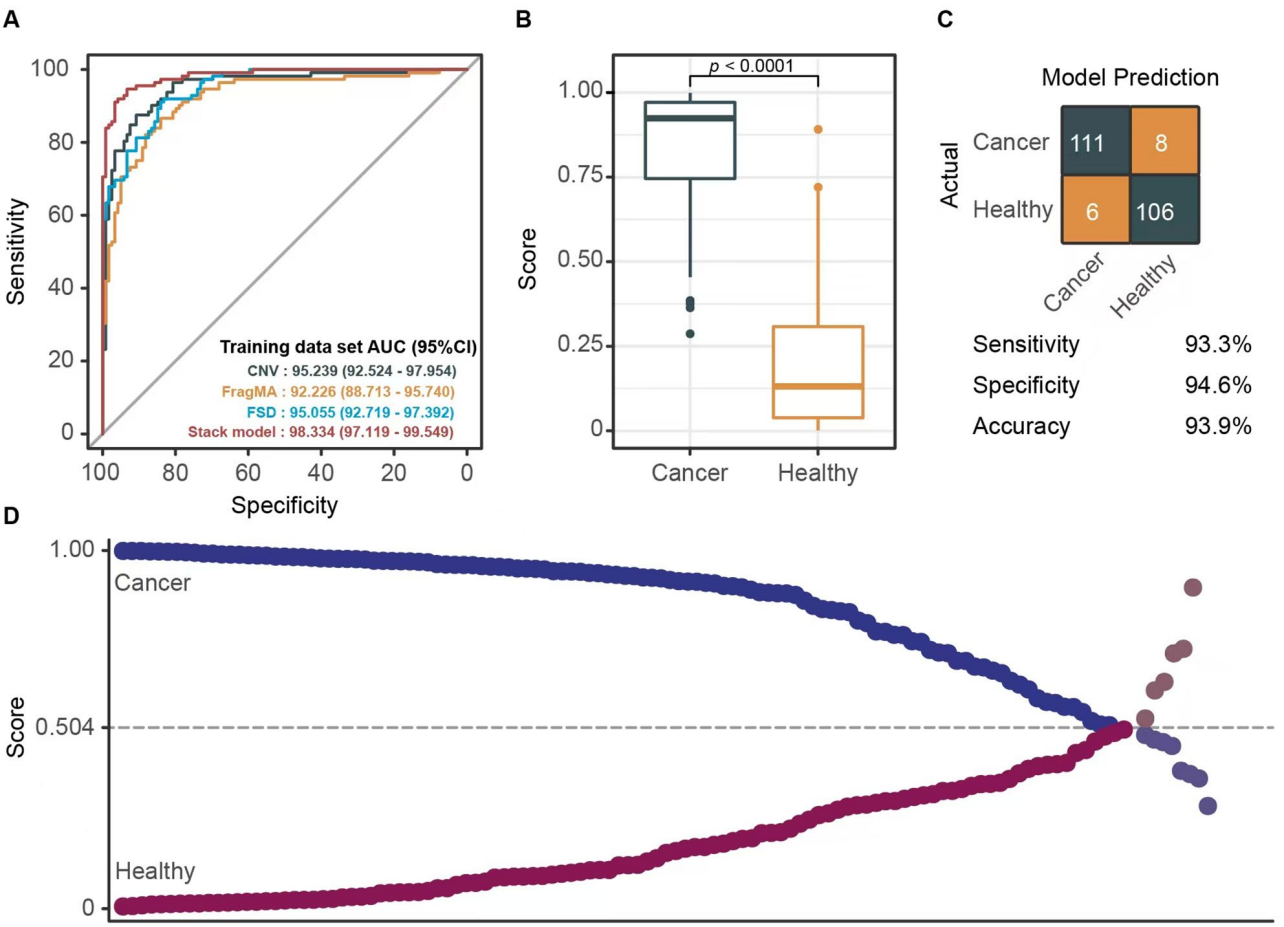
Model construction in training dataset. **A **Receiver operating characteristic (ROC) curves evaluating the model’s predictive performance of base model (CNV, FSD, FragMA) and stack model. **B **The median score of samples from breast cancer patients is significantly higher than that of healthy individuals, with a Wilcoxon test p-value of less than 0.01. **C **The confusion matrix, sensitivity, specificity, and accuracy of the ensemble stack prediction model in the training dataset. **D **Scatterplot showing the model prediction scores for breast cancer and healthy samples, as well as labelling the false detections under the 95% specificity cut-off (0.504).

Feature assessment and ensemble stacked model evaluationModel selection was conducted by assessing the AUC values obtained from various combinations of the cfDNA features and machine learning algorithms across the validation cohorts. We evaluated the performance of FRAGMA, FSD, and CNV features within all foundational models (including GLM, GBM, deep learning, random forest, and XGBoost) in terms of their AUC metrics. The analysis of different foundational models demonstrated that the algorithm-stacked model outperformed others, as the integration of FRAGMA, FSD, and CNV features led to higher AUC values within the stacked model compared to those from individual algorithm models. Consequently, we developed a stacked ensemble model that incorporates the three plasma cfDNA fragmentomic features (FRAGMA, FSD, and CNV) alongside six machine learning algorithms (XRT, GLM, GBM, random forest, deep learning, and XGBoost) (see Fig. 1B). The stacked ensemble model achieved an enhanced predictive capability, resulting in an AUC of 0.983, which surpassed the performance of stacked models that utilized individual fragmentomic features (Fig. 2A). Furthermore, the AUC of the stacked ensemble model exceeded that of a generalized linear model (GLM) based on fragmentomic features. Consequently, we selected the stacked ensemble model for subsequent evaluations. Our analysis revealed that the predicted cancer scores for cancer patients were significantly greater than those of healthy individuals within the training cohort (Fig. 2B). In the training set, the model demonstrated sensitivity and specificity rates of 93.3% and 94.6%, respectively, in differentiating breast cancer from healthy subjects (Fig. 2C). Figure 2D illustrates the prediction scores of the model for both breast cancer and healthy samples, additionally highlighting false detections that occurred under the 95% specificity threshold (0.504).

**Fig. 3:**
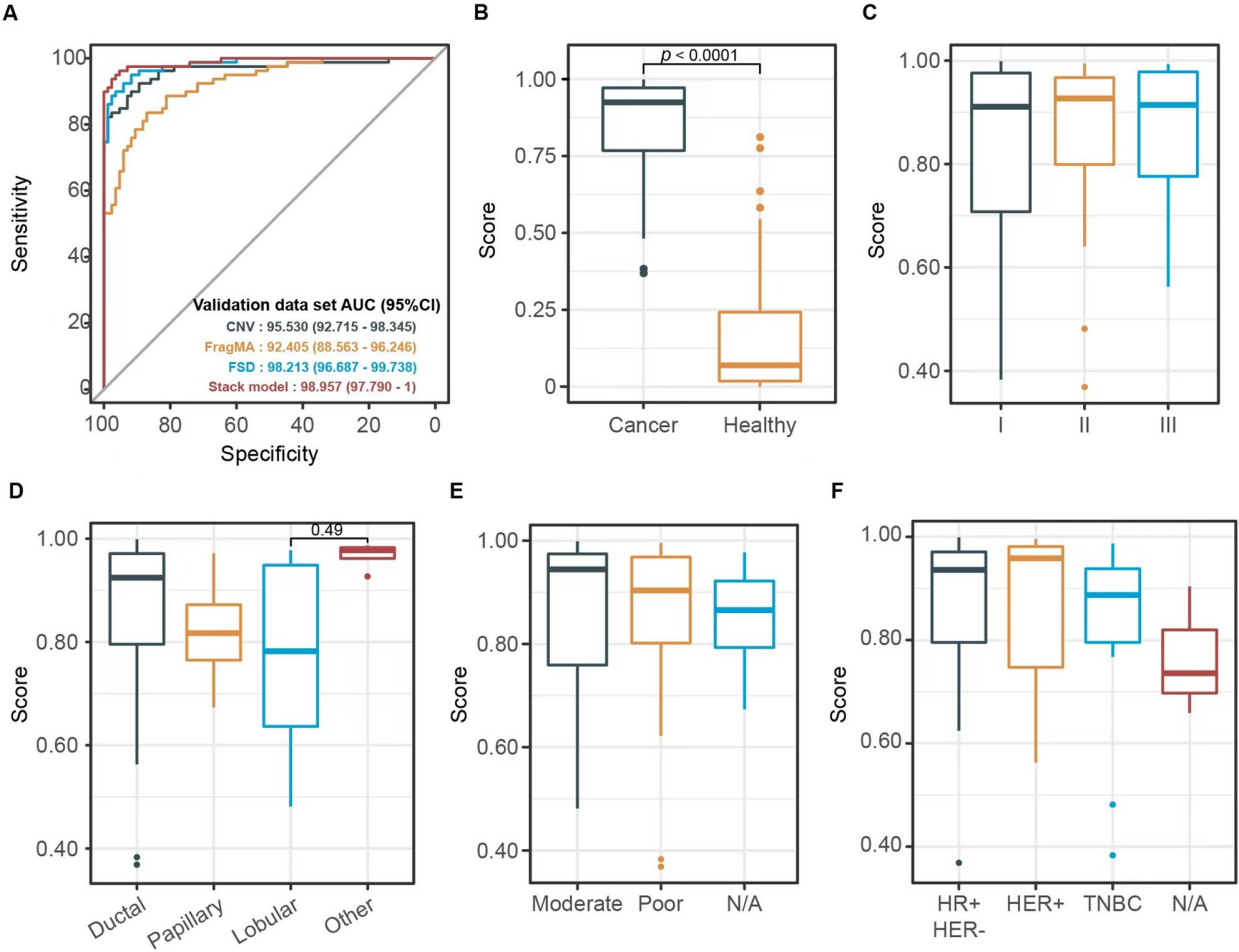
Model performance evaluation and subgroup analysis in validation dataset. **A** Receiver operating characteristic (ROC) curves evaluating the model’s predictive performance of base model (CNV, FSD, FragMA) and stack model. **B** The median score of samples from breast cancer patients is significantly higher than that of healthy individuals, with a Wilcoxon test p-value of less than 0.01 **C**-**F** Prediction score differences of breast cancer samples, stratified by histological subtype (**D**), differentiation level (**E**) and molecular classification (**F**)

We evaluated the effectiveness of the stacked ensemble model using the validation dataset. The area under the curve (AUC) of the model remains notably high, measuring 0.989 in the validation cohort (Fig. 3A). The median scores for samples collected from breast cancer patients are considerably greater compared to those from healthy individuals, yielding a Wilcoxon test p-value of under 0.01 (Fig. 3B).

### Performance of the predictive model in different cancer sample subgroups

The performance of the model was additionally assessed across various breast cancer subgroups utilizing the validation datasets. Notably, the subgroups exhibited no statistically significant differences among the various categories (Fisher’s exact test, P values exceeding 0.05). As illustrated in Fig. 3C-F, the sensitivity of assay detection remains consistently elevated among different patient subgroups with breast cancer. Specifically, our model demonstrated sensitivity across various stages, pathologic types, levels of differentiation, and molecular classifications. It was important to note that moderate differentiation corresponded to histological grades II and III, whereas low differentiation was identified as histological grade I.

## Discussion

Emerging as a non-invasive supplement or substitute for traditional tumor biopsies, liquid biopsies are gaining prominence [37–41]. In this research, we present a stacked ensemble model constructed from three cfDNA characteristics (FSD, CNV, FRAGMA) utilizing six distinct algorithms (GLM, GBM, DL, XRF, DRF, and XGBoost). This model serves as an ultra-sensitive and cost-effective non-invasive method for breast cancer detection. Furthermore, our findings indicate potential advantages in stage shift at diagnosis when applied in real-world scenarios within Chinese demographics. Our objective was to improve the early identification of breast cancer by developing a stacked ensemble machine learning model that integrates various cfDNA fragmentomic features, thereby achieving high sensitivity in differentiating individuals with early-stage breast cancer from those without the disease.

Our multidimensional assay leverages the advantages of cfDNA fragmentomic characteristics within a stacked ensemble machine learning framework. The cleavage and fragmentation of cfDNA occur in a non-random manner, with certain genomic regions exhibiting preferred cleavage patterns, known as preferred end sites. These patterns are associated with factors such as tissue origin and disease states, which are influenced by chromatin accessibility, nuclease activities, and other elements [42].Given that preferred end sites linked with tumors are more widespread and easily detected than mutations, fragmentomic features have emerged as a significant category of ctDNA signatures [42]. The exceptional performance of our model underscores the potential of cfDNA fragmentomics in cancer detection. As previously mentioned, patterns of cfDNA methylation and fragmentation have been employed to identify breast cancer patients; however, their sensitivity for clinical application remains suboptimal [14, 42–45]. We have conducted a comprehensive assessment of the current characteristics of circulating free DNA (cfDNA) and integrated the most effective attributes into our predictive model. Ensemble machine learning models exhibit significant advantages in terms of accuracy, reproducibility, and interpretability for bioinformatics applications. Additionally, the stacking approach effectively consolidates the predictions produced by individual base models [44].

Focusing exclusively on the whole genome sequencing (WGS) of plasma cell-free DNA (cfDNA), our research presents an exemplary performance model for cancer identification. It attains an area under the curve (AUC) of 0.9833 in the independent validation cohort and reaches an AUC of 0.9895 in prospective evaluations. This remarkable predictive capability is due to a comprehensive ensemble stacked model utilizing machine learning techniques and relying on three cfDNA characteristics. Importantly, no individual-feature base model was able to achieve comparable outcomes. The ensemble stacked model’s error correction features improve overall predictive accuracy (for instance, accurately classifying non-cancer individuals as non-cancer and cancer individuals as cancer). This advancement leads to heightened sensitivity and specificity, yielding a superior AUC in comparison to the base models. Furthermore, it is important to note that this model exhibits strong performance across various subgroups, including tumor stage, histological grade, and molecular classification.

This study highlights several limitations. While we have demonstrated the application of cfDNA fragmentomics for enhanced sensitivity in detecting breast cancer, the underlying mechanisms in this field remain poorly understood. We are currently conducting an extensive inquiry aimed at identifying factors and revealing new characteristics that could refine our model. Furthermore, the sample size is somewhat limited, which may lead to an imprecise evaluation of sensitivity within smaller subgroups, including tumors with low or high differentiation. Moreover, we were unable to include a fully external cohort from independent institutions in the current analysis. We recognize that such external validation is essential to firmly establish clinical utility and minimize potential biases. Moving forward, we are actively planning a prospective, multi-center study to evaluate the assay’s performance in a real-world screening context.

## Conclusions

In conclusion, we created a stacked ensemble model that integrates three fragmentomic characteristics obtained from plasma cfDNA whole genome sequencing (WGS) data. Our model showed remarkable sensitivity in distinguishing patients with early-stage breast cancer from those without the condition. In addition to improving the ability to detect very early and small tumors in breast cancer patients, our comprehensive model provides an accurate and economical approach for promoting early breast cancer detection and improving patient outcomes.

## Supplementary Information

Below is the link to the electronic supplementary material.

Supplementary Material 1

Supplementary Material 2

Supplementary Material 3

Supplementary Material 4

## Data Availability

The data that support the findings of this study are available from the corresponding author upon reasonable request.
