# Supplementary figures and images for: Multidimensional cell-free DNA fragmentomics enables early detection of breast cancer
Source: Breast Cancer Res. 2025 Dec 9;28:6. doi: 10.1186/s13058-025-02190-8 (PMC12801790; doi:10.1186/s13058-025-02190-8)

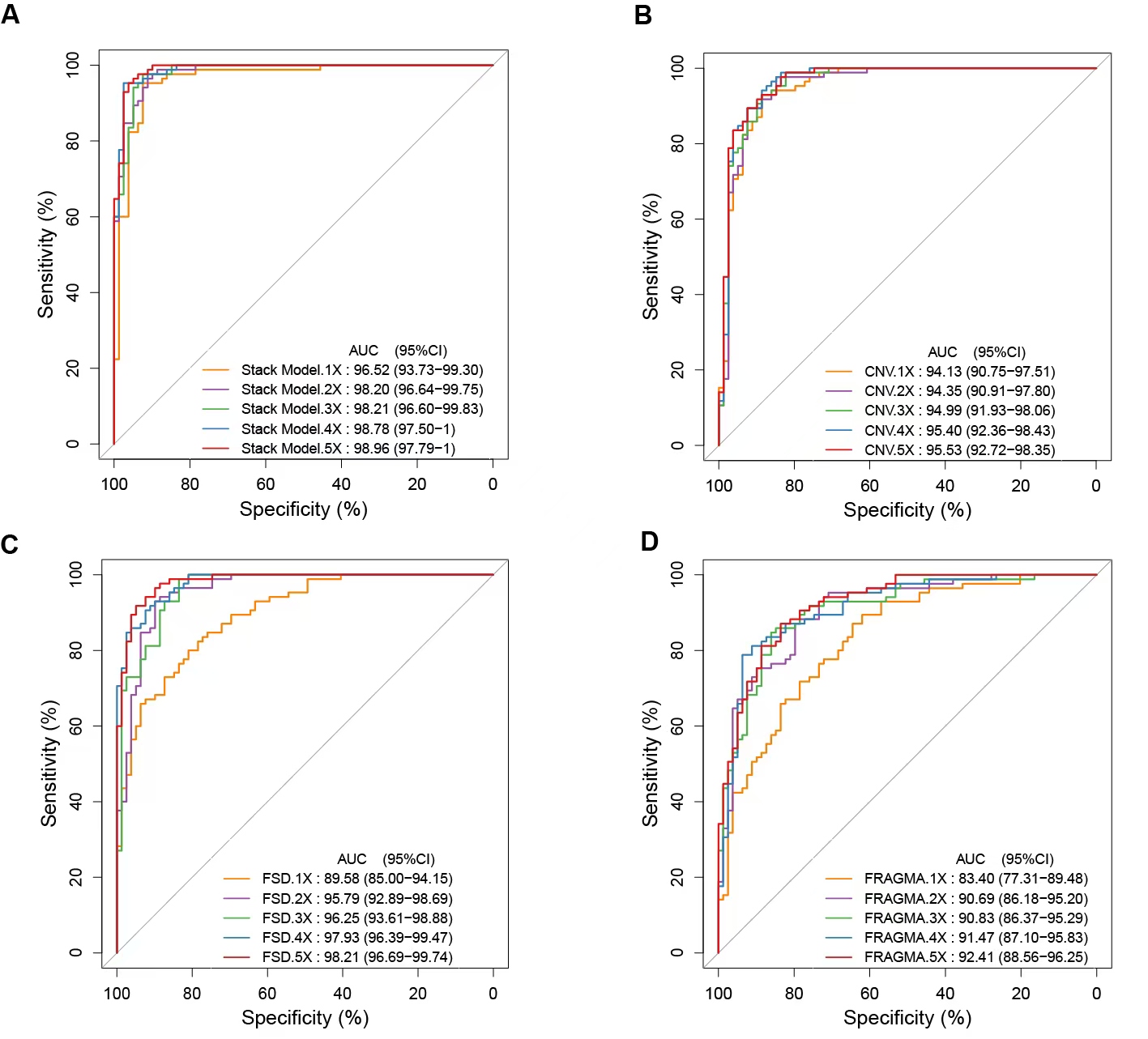

Supplement: Supplementary file 1 — Supplementary Material 1 [file 13058_2025_2190_MOESM1_ESM.jpg]
