# Supplementary material for: Multidimensional cell-free DNA fragmentomics enables early detection of breast cancer
Source: Breast Cancer Res. 2025 Dec 9;28:6. doi: 10.1186/s13058-025-02190-8 (PMC12801790; doi:10.1186/s13058-025-02190-8)
Supplement: Supplementary file 2 — Supplementary Material 2 [file 13058_2025_2190_MOESM2_ESM.docx]

Supplementary Figure 1. Variation in prediction scores for cancer and healthy samples within the training set as sequencing depth is downsampled from 5X to 4X, 3X, 2X and 1X.
