# Supplementary material for: Multidimensional cell-free DNA fragmentomics enables early detection of breast cancer
Source: Breast Cancer Res. 2025 Dec 9;28:6. doi: 10.1186/s13058-025-02190-8 (PMC12801790; doi:10.1186/s13058-025-02190-8)
Supplement: Supplementary file 4 — Supplementary Material 4 [file 13058_2025_2190_MOESM4_ESM.docx]

Supplementary Table 1 legend: Performance metrics of each individual feature model for both training and validation set
